# Bile Acid-Induced Virulence Gene Expression of *Vibrio parahaemolyticus* Reveals a Novel Therapeutic Potential for Bile Acid Sequestrants

**DOI:** 10.1371/journal.pone.0013365

**Published:** 2010-10-13

**Authors:** Kazuyoshi Gotoh, Toshio Kodama, Hirotaka Hiyoshi, Kaori Izutsu, Kwon-Sam Park, Rikard Dryselius, Yukihiro Akeda, Takeshi Honda, Tetsuya Iida

**Affiliations:** 1 Laboratory of Genomic Research on Pathogenic Bacteria, International Research Center for Infectious Diseases, Research Institute for Microbial Diseases, Osaka University, Osaka, Japan; 2 Department of Bacterial Infections, Research Institute for Microbial Diseases, Osaka University, Osaka, Japan; 3 Department of Food Science and Bio Technology, College of Ocean Science and Technology, Kunsan National University, Kunsan, Korea; 4 Department of Cell and Molecular Biology, Karolinska Institutet, Stockholm, Sweden; 5 Laboratory of Clinical Research on Infectious Disease, International Research Center for Infectious Diseases, Research Institute for Microbial Diseases, Osaka University, Osaka, Japan; Columbia University, United States of America

## Abstract

*Vibrio parahaemolyticus*, a bacterial pathogen, causes human gastroenteritis. A type III secretion system (T3SS2) encoded in pathogenicity island (Vp-PAI) is the main contributor to enterotoxicity and expression of Vp-PAI encoded genes is regulated by two transcriptional regulators, VtrA and VtrB. However, a host-derived inducer for the Vp-PAI genes has not been identified. Here, we demonstrate that bile induces production of T3SS2-related proteins under osmotic conditions equivalent to those in the intestinal lumen. We also show that bile induces *vtrA*-mediated *vtrB* transcription. Transcriptome analysis of bile-responsive genes revealed that bile strongly induces expression of Vp-PAI genes in a *vtrA*-dependent manner. The inducing activity of bile was diminished by treatment with bile acid sequestrant cholestyramine. Finally, we demonstrate an *in vivo* protective effect of cholestyramine on enterotoxicity and show that similar protection is observed in infection with a different type of *V. parahaemolyticus* or with non-O1/non-O139 *V. cholerae* strains of vibrios carrying the same kind of T3SS. In summary, these results provide an insight into how bacteria, through the ingenious action of Vp-PAI genes, can take advantage of an otherwise hostile host environment. The results also reveal a new therapeutic potential for widely used bile acid sequestrants in enteric bacterial infections.

## Introduction

*Vibrio parahaemolyticus* is a Gram-negative marine bacterium responsible for acute gastroenteritis associated with the consumption of raw or undercooked contaminated seafood [1]. *V. parahaemolyticus* infection is a growing public health concern because of the emergence of pandemic strains that have caused severe outbreaks worldwide [2], [3].

Most *V. parahaemolyticus* strains isolated from clinical cases exhibit hemolytic activity on a special blood agar called Wagatsuma agar [4]. This hemolysis, termed the Kanagawa phenomenon (KP), has been considered a good virulence marker of pathogenic *V. parahaemolyticus* [5]. The KP is caused by thermostable direct hemolysin (TDH) produced by this bacterium [6]. As purified TDH has multiple biological activities, including induction of fluid accumulation in the rabbit intestine, it has been considered a major virulence factor of *V. parahaemolyticus* [6]–[16].

Since these studies, whole genome sequencing of a KP-positive *V. parahaemolyticus* strain revealed the presence of two sets of type III secretion systems: T3SS1 and T3SS2 [17]. Comparative genome analysis using microarrays showed that an 80 kb pathogenicity island (Vp-PAI) on chromosome II, which encodes two *tdh* genes and the T3SS2 gene cluster, is unique to KP-positive pathogenic strains [18], [19]. According to a recent report that evaluated the fluid-accumulating activity of virulence gene deletion mutants in a rabbit ileal loop model, T3SS2, not TDH or T3SS1, contributes to *V. parahaemolyticus*-induced enterotoxicity [20].

T3SS gene clusters similar to the T3SS2 of KP-positive *V. parahaemolyticus* have been detected in both TDH-related hemolysin (*trh*)-positive (KP-negative) *V. parahaemolyticus* and non-O1/non-O139 *V. cholerae*, which are also pathogenic to humans [21], [22]. The T3SS2-related genes of *trh*-positive *V. parahaemolyticus* are involved in enterotoxicity [22], whereas those of non-O1/non-O139 *V. cholerae* appear to be required for intestinal colonization [21]. Consequently, the T3SSs are believed to be pivotal for the pathogenicity of these bacteria.

Recently, we reported that two ToxR-like proteins, VtrA and VtrB, specifically regulate the transcription of genes encoded within Vp-PAI, including the genes for TDH and the T3SS2-related proteins. Because null mutants of *vtrA* and *vtrB* did not exhibit any *in vivo* fluid-accumulating activity in the rabbit intestine, this demonstrated that these regulators play critical roles in the enterotoxicity of *V. parahaemolyticus* [23]. Although this shows that expression of Vp-PAI genes is required for induction of fluid accumulation in the intestine, the environmental and/or host-derived factors that trigger the expression of these genes remain unknown. The aim of this study was to identify a host factor that triggers the expression of Vp-PAI genes and to determine its role in the pathogenicity of *V. parahaemolyticus*.

## Results

### Crude bile stimulates the production of TDH and T3SS2-related proteins under intestinal osmotic conditions

**Figure 1:**
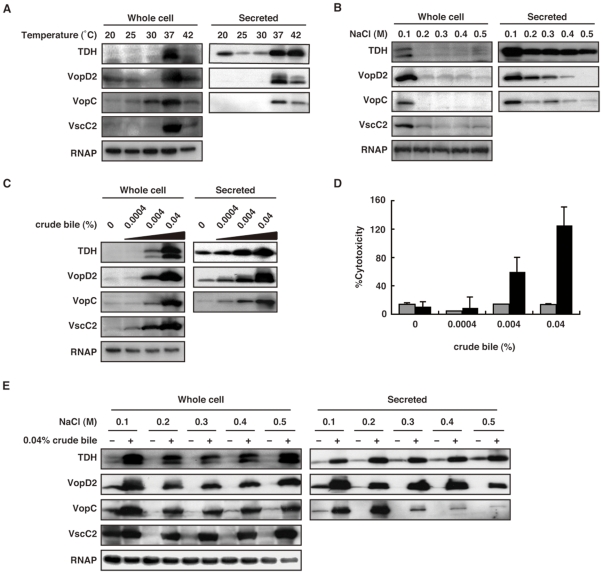
Crude bile stimulates the production of TDH and T3SS2-related protein under high osmotic conditions. A. Effect of temperature on the production of TDH and T3SS2-related proteins. Immunoblot analysis of bacterial whole cell pellets (left) and secreted proteins (right) from WT *V. parahaemolyticus* cultured in LB medium containing 0.5% NaCl at various temperatures (20–42°C). Blots were probed with anti-TDH, anti-VopD2, anti-VopC, anti-VscC2 and anti-RNAP antibodies. B. Effect of osmolarity of the culture medium on the production of TDH and T3SS2-related proteins. Immunoblot analysis of bacteria cultured in LB medium containing various concentrations of NaCl (0.1–0.5 M) at 37°C. C. Effect of crude bile on the production of TDH and T3SS2-related proteins. Immunoblot analysis of bacteria cultured in LB medium containing 0.3 M NaCl at 37°C in the presence of various concentrations of crude bile (0–0.04%). D. Crude bile promoted the T3SS2-dependent cytotoxicity of *V. parahaemolyticus*. *V. parahaemolyticus* strains (Δ*vcrD1*Δ*vcrD2* [TDH and T3SS1/T3SS2-deficient strain], gray bar; POR-2 [TDH and T3SS1-deficient strain], black bar) were cultured in LB medium containing 0.3 M NaCl at 37°C in the presence of various concentrations of crude bile (0–0.04%) for 3 h. After incubation, bacteria were used for infection of Caco-2 cells for 4.5 h. Cytotoxicity was evaluated according to the amount of LDH released. Error bars represent the SDs of means from triplicate independent experiments. E. Crude bile stimulation overcomes the repression of TDH and T3SS2-related protein production under high osmotic pressure. Immunoblot analysis of *V. parahaemolyticus* cultured in LB medium containing various concentrations of NaCl (0.1–0.5 M) in the presence (+) or absence (−) of 0.04% crude bile.

To identify an environmental factor in the intestine that affects the expression of Vp-PAI genes, we first examined the effect of cultivation temperature on the production of TDH and the T3SS2-related proteins, VopD2 (T3SS2 translocon protein), VopC (T3SS2 effector protein) and VscC2 (T3SS2 apparatus protein), using immunoblotting. Much higher abundances of these proteins were detected when bacteria were cultured at 37 and 42°C, which corresponds to the temperature of the intestine, than at lower temperatures (Fig. 1A). To determine the effect of extracellular osmotic pressure on the production of these proteins, we next grew *V. parahaemolyticus* at 37°C in LB medium and adjusted the osmotic pressure from 0.1 M to 0.5 M by adding NaCl (Fig. 1B). The greatest production of Vp-PAI proteins was observed for bacteria cultured in medium containing 0.1 M NaCl. Protein production gradually decreased as NaCl concentration increased and was particularly impaired at NaCl concentrations greater than 0.3 M. This result was unexpected because the osmolarity of the intestinal lumen is estimated to exceed that of a 0.3 M NaCl solution [24]. Therefore, we reasoned that a host-derived inducer (or host-derived inducers) that counteracts the inhibitory effect of intestinal osmotic pressure on Vp-PAI protein production must exist in the intestinal tract. After evaluating various candidate factors, we finally identified bile as a potent stimulator of the production of these proteins. As shown in Fig. 1C, crude bile induced the production of TDH and T3SS2-related proteins in a concentration-dependent manner in the presence of 0.3 M NaCl. The inducing activity of crude bile became saturated at a concentration of 0.04%, which corresponds with the concentration of bile in the intestinal tract. A characteristic of pathogenic *V. parahaemolyticus* is its T3SS2-mediated cytotoxic effects on Caco-2 cells *in vitro* [25]. Therefore, the effect of crude bile on cytotoxicity was evaluated by preculturing bacteria in the presence or absence of various concentrations of crude bile (Fig. 1D). Although a TDH- and T3SS1-deficient strain (POR-2) precultured without crude bile did not induce any apparent cytotoxic effects, crude bile stimulated the cytotoxicity of this strain in a concentration-dependent manner. No cytotoxic effect was observed in a TDH- and T3SS1/T3SS2-deficient strain (Δ*vcrD1/*Δ*vcrD2*) regardless of the presence of bile. To ensure that the stimulatory effect of bile was specific for T3SS2 and TDH, the effect of crude bile on the production of T3SS1-related proteins was also examined (Fig. S1A). In contrast to the aforementioned crude bile-mediated increase in the production of T3SS2-related proteins and TDH, with this strain an increase in the concentration of crude bile was accompanied by a decrease in the production of T3SS1-related proteins. In addition, this repressive effect of crude bile was reflected in decreased T3SS1-dependent cytotoxicity (Fig. S1B). As the osmolarity of the intestinal lumen is higher than that of a 0.3 M NaCl solution, we next determined whether bile-induced TDH- and T3SS2-protein expression would overcome the repressive effects of high osmotic pressure. Fig. 1E shows that crude bile stimulated the production of these proteins at concentrations of up to 0.5 M NaCl. Together, these results indicate that crude bile is a potent host-derived inducer of TDH and T3SS2-related protein production under osmotic conditions corresponding to those in the intestinal tract.

### VtrA and VtrB mediate crude bile-induced production of T3SS2-related proteins and TDH

**Figure 2:**
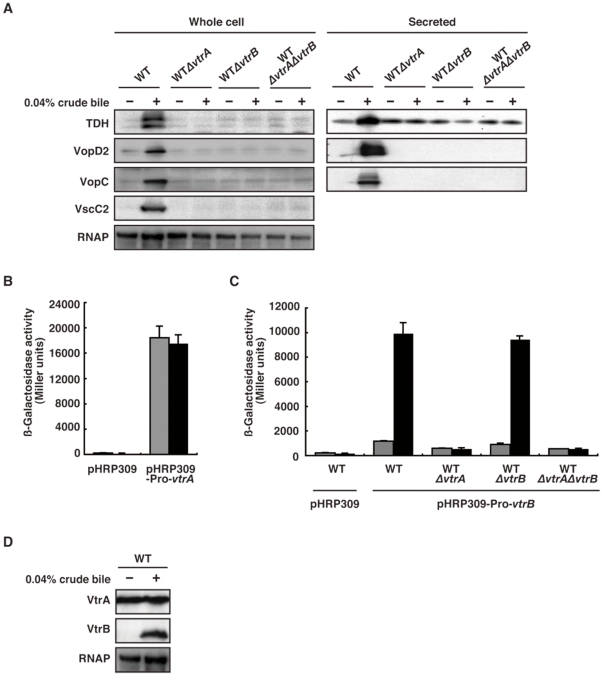
VtrA and VtrB are required for the production of crude bile-induced T3SS2-related proteins and TDH. A. Loss of *vtrA* and/or *vtrB* diminishes crude bile-induced TDH and T3SS2-related protein production. Western blot analysis of bacterial pellets (left panel) and secreted proteins (right panel) from isogenic mutants of WT *V. parahaemolyticus* cultured in the presence (+) or absence (−) of 0.04% crude bile. Blots were probed with RNAP, TDH and T3SS2-related antibodies. B. Crude bile stimulation does not affect the transcription of *vtrA*. *V. parahaemolyticus* strains carrying a control vector (left) or a *vtrA-lacZ* transcription fusion vector (right) were cultured in the absence (gray bars) or presence (black bars) of 0.04% crude bile. β-Galactosidase activity is expressed in Miller units. Data are expressed as the average ± SD of three separate experiments. C. Crude bile stimulates *vtrA*-mediated *vrtB* transcription. *V. parahaemolyticus* strains carrying a control vector or a *vtrB-lacZ* transcription fusion vector were cultured in the absence (gray bars) or presence (black bars) of 0.04% crude bile. β-Galactosidase activity is expressed in Miller units. Data are expressed as the average ± SD of three separate experiments. D. Crude bile induces the production of VtrB protein. Immunoblots of WT *V. parahaemolyticus* cultured in the presence (+) or absence (−) of 0.04% crude bile. Blots were probed with anti-VtrA (upper panel), anti-VtrB (middle panel) and anti-RNAP (lower panel) antibodies.

It has been reported that three transcriptional regulators are involved in the expression of TDH and/or T3SS2-related proteins. One of these, Vp-ToxR, shares some identity with the *V. cholerae* ToxR (Vc-ToxR). The Vc-ToxR is known to regulate expression of multiple genes, including the cholera toxin (CT) and toxin-coregulated pilus (TCP) genes [26], and is also involved in sodium cholate induction of CT [27]. The other candidates are VtrA and VtrB, which were recently identified as master regulators of virulence gene expression in the Vp-PAI [23]. We next examined whether any of these regulators are involved in bile-induced production of TDH and T3SS2-related proteins. No participation of the Vp-ToxR gene in crude bile-induced production of TDH and T3SS2-related proteins was observed (Fig. S2A). A WTΔ*toxR* strain induced fluid accumulation in a rabbit ileal loop to a similar extent as WT bacteria over a range of inoculation doses (Fig. S2B). In contrast to Vp-ToxR, *vtrA* and *vtrB* deletion mutants did not induce any fluid accumulation (Fig. S2B), nor did they produce TDH and T3SS2-related proteins, even in the presence of crude bile (Fig. 2A). As a previous report showed that the expression of VtrB is controlled directly by VtrA [23], we next examined whether crude bile affects the expression of these regulators using *vtrA-lacZ* and *vtrB-lacZ* transcriptional fusion reporters. As shown in Fig. 2B, crude bile stimulation did not have any influence on *vtrA-lacZ* transcription. In contrast, substantial induction of *vtrB-lacZ* transcription by crude bile was observed in both WT and *vtrB*-deficient *V. parahaemolyticus* strains (WT and WTΔ*vtrB*), whereas deletion of the *vtrA* gene (WTΔ*vtrA*) caused a lack of responsiveness similar to that of a double deletion mutant (WTΔ*vtrA*Δ*vtrB*) (Fig. 2C). Immunoblotting of VtrA and VtrB revealed that the production of VtrA was constant regardless of the presence of crude bile, whereas the production of VtrB protein was induced only when crude bile was present (Fig. 2D). These results indicate that crude bile induces VtrA-mediated VtrB expression and that this transcriptional regulatory cascade is essential for crude bile-induced production of TDH and T3SS2-related proteins.

### Genome-wide transcriptional analysis of crude bile-responsive genes in *V. parahaemolyticus*

**Figure 3:**
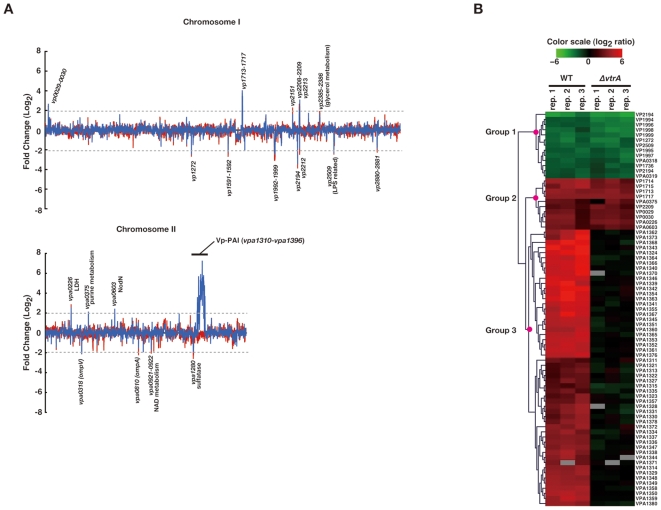
Genome-wide transcriptional analysis of crude bile-responsive genes in *V. parahaemolyticus*. A. Gene expression was determined by comparing cDNA generated from WT (blue line) or a *vtrA* mutant strain (red line) grown in the presence of 0.04% crude bile with that generated from the WT grown in the absence of crude bile. Data are expressed as the average of triplicate experiments. The Vp-PAI (VPA1310-VPA1369) region on chromosome 2 is indicated by a bold line. Gray dotted lines indicate the boundaries for a fourfold change and the identities of genes that were significantly upregulated or downregulated (≥ fourfold difference, P*<*0.05) in response to crude bile in the WT strain are indicated. B. Hierarchical cluster plot showing the relative expression of *V. parahaemolyticus* genes that were upregulated or downregulated in the presence of crude bile. Green indicates repression and red indicates induction of at least fourfold relative to the untreated samples. The scale bar indicates the mean of the log ratio. Gene identities are listed on the right.

For complete identification of the crude bile-responsive genes of *V. parahaemolyticus*, genome-wide transcriptional profiles of WT or *vtrA*-deficient strains grown in the presence of 0.04% crude bile were compared with that of the WT strain grown in the absence of crude bile (Fig. 3). The expression levels of 77 genes displayed significant changes (≥ a fourfold difference, P<0.05) in WT cells grown in the presence of crude bile (Table S1). In most cases, upregulation was observed and, interestingly, most of the upregulated genes were located within a distinct region of chromosome 2 (Fig. 3A). This region is included in the Vp-PAI region; in the *vtrA*-deficient strain, induction of these genes was absent (Table S1). Hierarchical clustering analysis classed the genes of filtered microarray data into three clusters: genes downregulated by crude bile stimulation in both WT and WTΔ*vtrA* (Group1), genes upregulated in both WT and WTΔ*vtrA* (Group2) and genes upregulated in WT but not significantly changed in WTΔ*vtrA* (Group3) (Fig. 3B). Interestingly, all the genes in Group3 were encoded within the Vp-PAI region. These results indicate that crude bile specifically promotes expression of Vp-PAI genes and that this induction is regulated by *vtrA*.

### Identification of a transcription-inducing substance for Vp-PAI genes in crude bile

**Figure 4:**
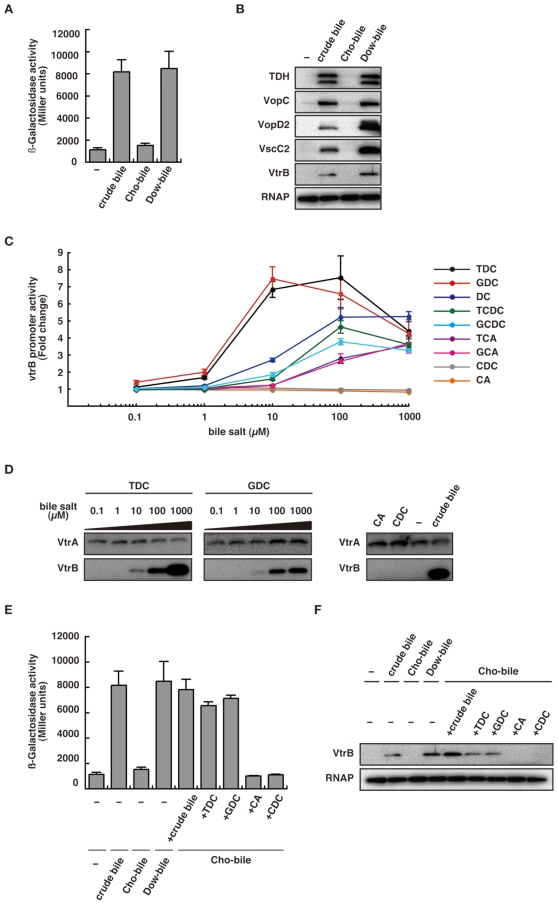
Identification of a transcriptional inducer for Vp-PAI genes in crude bile. A. *VtrB* expression in bile acid-sequestered crude bile. *V. parahaemolyticus* carrying the *vtrB-lacZ* transcription fusion vector were incubated in LB medium (0.3 M NaCl) with no crude bile (−), crude bile, cholestyramine-treated crude bile (Cho-Bile), or Dowex-treated crude bile (Dow-Bile). β-Galactosidase activity is expressed in Miller units. Data are expressed as the average ± SD of three separate experiments. B. Bile acid depletion from crude bile diminishes induction of TDH and T3SS2-related protein production. Immunoblot analysis of *V. parahaemolyticus* cultured in LB broth containing 0.3 M NaCl at 37°C with no crude bile (−), crude bile, cholestyramine-treated crude bile (Cho-Bile) or Dowex-treated crude bile (Dow-Bile). Blots were probed with anti-TDH, anti-VopD2, anti-VopC, anti-VscC2, anti-VtrB and anti-RNAP (RNA polymerase) antibodies. C. VtrB expression in the presence of various bile salts. *vtrB-lacZ* transcriptional activity was measured in the presence of various concentrations (0.1–1000 µM) of taurodeoxycholate (TDC; black line), glycodeoxycholate (GDC; red line), deoxycholate (DC; blue line), taurochenodeoxycholate (TCDC; green line), glycochenodeoxycholate (GCDC; light blue line), taurocholate (TCA; purple line), glycocholate (GCA; pink line), chenodeoxycholate (CDC; gray line) or cholate (CA; orange line). The data are expressed as the average ± SD of the ratio of LacZ activity in cells grown with bile acids relative to LacZ activity in cells grown without bile acids for three separate experiments. D. TDC and GDC independently induce the production of VtrB protein. Immunoblot analysis of *V. parahaemolyticus* cultured in LB medium containing 0.3 M NaCl at 37°C with various concentrations (0.1–1000 µM) of TDC (left panel) or GDC (middle panel) or with 1000 µM of CA, CDC or with 0.04% crude bile (right panel). Blots were probed with anti-VtrA and anti-VtrB antibodies. E. The addition of TDC or GDC restores the diminished activity of cholestyramine-treated crude bile to induce *vtrB* expression. Cholestyramine-treated crude bile (Cho-bile) was supplied with 0.04% crude bile or 100 µM of TDC, GDC, CA or CDC. Effects on *vtrB* expression were assessed according to a β-galactosidase assay. β-Galactosidase activity is expressed in Miller units. Data are expressed as the average ± SD of three separate experiments. F. Effect of the addition of bile acids to cholestyramine-treated crude bile on the production of VrtB protein. Cholestyramine-treated crude bile (Cho-bile) was supplied with 100 µM of TDC, GDC, CA or CDC. Effects on VtrB production were assessed according to immunoblotting using anti-VtrB and anti-RNAP antibodies.

Crude bile is a mixture of organic and inorganic compounds whose major constituents include bile acids, cholesterol, phospholipids and the pigment biliverdin [28]. To identify a transcription-inducing substance for Vp-PAI genes in crude bile, we next examined the effect of bile acid depletion. The ability of crude bile to induce *vtrB-lacZ* transcription disappeared upon treatment with bile acid sequestered with cholestyramine resin (Cho-bile), whereas no such effect was observed after treatment with the control resin, Dowex800 400 mesh (Dow-bile), which does not bind bile acids [29] (Fig. 4A). Similar results were observed for immunoblotting of TDH and T3SS2-related proteins, as the cholestyramine-treated crude bile lacked the capacity to induce them (Fig. 4B). These results suggest that bile acids are essential for inducing transcription of Vp-PAI genes. We then examined the ability of nine individual bile salts that are present in the intestinal tract to induce *vtrB* expression. The results (Fig. 4C) revealed that these bile salts could be classed into three groups with respect to their transcription-inducing activity: those with high inducing activity (taurodeoxycholate, TDC; and glycodeoxycholate, GDC), those with intermediate inducing activity (deoxycholate, DC; taurochenodeoxycholate, TCDC; glycochenodeoxycholate, GCDC; taurocholate, TCA; and glycocholate, GCA) and those lacking inducing activity (chenodeoxycholate, CDC; and cholate, CA). These results were confirmed by immunoblotting analysis of bile salts that had the highest level of VtrB induction and those that had no VtrB-inducing activity (Fig. 4D). In addition, the extent to which individual bile salts induced *vtrB* expression was strongly correlated with the level of production of TDH and T3SS2-related proteins; TDC and GDC were the strongest inducers, whereas CDC and CA lacked inducing activity (Fig. S3A and B). Therefore, these four bile salts were used in subsequent experiments. To confirm that the loss of the ability of cholestyramine-treated crude bile to induce Vp-PAI genes was caused by depletion of bile acids, we examined the effect of addition of supplementary bile salts to cholestyramine-treated crude bile on *vtrB-lacZ* transcriptional activity (Fig. 4E). The diminished ability of cholestyramine-treated crude bile to induce *vtrB-lacZ* transcription was fully restored not only by addition of crude bile but also by the addition of TDC or GDC, whereas neither CDC nor CA addition overcame the loss of induction activity. Similar results were obtained for VtrB protein immunoblotting: production of VtrB was induced only when crude bile, TDC or CA were added to cholestyramine-treated crude bile (Fig. 4F). These results suggest that bile acids in crude bile, especially TDC and GDC, are major transcription-inducing substances for Vp-PAI genes.

### Endogenous bile acids in the intestinal tract of rabbits are necessary for induction of fluid accumulation

**Figure 5:**
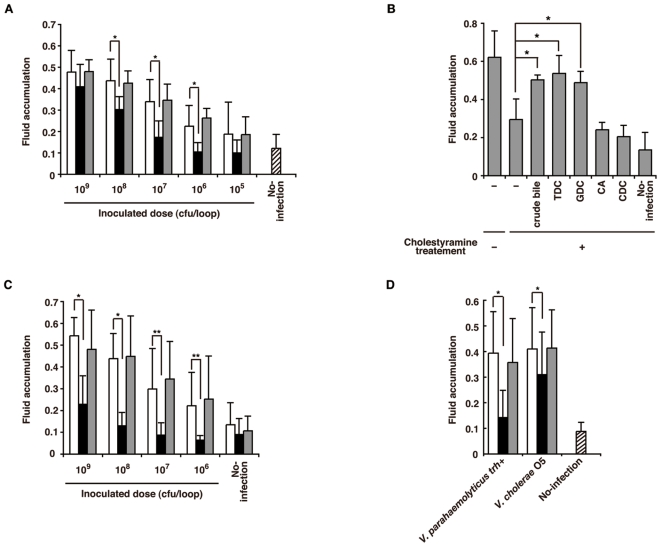
Endogenous bile acids in the intestinal tract are necessary for efficient induction of fluid accumulation by *V. parahaemolyticus*. A. Absorptive removal of endogenous bile salts from the intestinal tract using cholestyramine attenuates *V. parahaemolyticus*-induced fluid accumulation. Rabbit ileal loops were treated with PBS (white bars), 5% cholestyramine (black bars) or 5% Dowex800 400 mesh (gray bars) before washing with PBS and infection with various doses of bacteria (10^5^–10^9^ CFU). Fluid accumulation in each loop was measured 16 h after the challenge. Values are expressed in terms of the amount of accumulated fluid (ml) per cm of ligated rabbit small intestine. Error bars represent SDs for experiments conducted in sextuplicate. Asterisks indicate significant differences from the PBS-treated ileal loops (P*<*0.001). B. Addition of TDC or GDC restores the attenuated fluid accumulation of cholestyramine-treated ileal loops. *V. parahaemolyticus* (10^7^ CFU) were suspended in LB medium containing 0.04% crude bile or 100 µM TDC, GDC, CA or CDC before injection into cholestyramine-treated ileal loops. The fluid accumulation in each loop was measured 16 h after infection. Error bars represent SDs for means from experiments conducted in sextuplicate. Asterisks indicate significant differences from nonsupplemented control loops (P*<*0.05). C. Prevention of *V. parahaemolyticus*-induced fluid accumulation by coadministration of cholestyramine. Various doses of *V. parahaemolyticus* (10^6^–10^9^ CFU) were suspended in LB medium alone (white bars), LB medium containing 1% cholestyramine (black bars) or LB medium containing 1% Dowex800 400 mesh (gray bars) before injection into rabbit ileal loops. Fluid accumulation in each loop was measured 16 h after infection. Error bars represent SDs for experiments conducted in sextuplicate. Asterisks indicate significant differences from the means for resin-free ileal loops (*P*<*0.001, **P*<*0.05). D. Prevention of *trh*-positive *V. parahaemolyticus*- and non-O1/non-O139 *V. cholerae*-induced fluid accumulation by coadministration of cholestyramine. TH3996 (*trh*-positive *V. parahaemolyticus*) or RIMD2214243 (non-O1/non-O139 *V. cholerae*) (10^9^ CFU) were suspended in LB medium alone (white bars), LB medium containing 1% cholestyramine (black bars) or LB medium containing 1% Dowex800 400 mesh (gray bars) before injection into rabbit ileal loops. Fluid accumulation in each loop was measured 16 h after infection. Error bars represent SDs for experiments conducted in sextuplicate. Asterisks indicate significant differences from resin-free ileal loops (*P*<*0.05).

To determine whether the presence of endogenous bile acids in the intestine is necessary for *in vivo* enterotoxicity, fluid accumulation induced by various doses of *V. parahaemolyticus* in cholestyramine-treated rabbit ileal loops were compared with fluid accumulation in untreated loops (Fig. 5A). Cholestyramine-treated rabbit ileal loops were prepared by inoculating the resin into the loops. The loops were then washed with PBS to remove residual resin before infection (see Material and methods). Although fluid accumulation in cholestyramine-treated loops inoculated with 10^9^ CFU of *V. parahaemolyticus* was similar to that in untreated loops, cholestyramine significantly reduced fluid accumulation at inoculation doses ranging from 10^6^ to 10^8^ CFU. In contrast, fluid accumulation in control resin (Dowex800 400 mesh)-treated loops was similar to that in untreated loops for every inoculation dose. To determine whether the attenuating effect of cholestyramine treatment on fluid accumulation was caused by absorptive removal of endogenous bile acids from the intestinal tract, we examined the effect of adding crude bile or individual bile salts to cholestyramine-treated loops (Fig. 5B). Fluid accumulation in cholestyramine-treated loops was significantly increased by the addition of crude bile or the *vtrB*-inducing bile salts, TDC and GDC, whereas addition of CA and CDC, which did not induce *vtrB* expression, did not increase fluid accumulation. As it was reported that norepinephrine—a hormone that exerts a modulatory effect in the gastrointestinal tract—enhances T3SS2-dependent fluid accumulation in a rat ileal loop model (Nakano et al., 2007), we examined whether the attenuating effect of cholestyramine-treatment on fluid accumulation was due to absorptive removal of norepinephrine. However, norepinephrine addition did not restore the reduced fluid accumulation of cholestyramine-treated loops (Fig. S4). We next examined the effect of coadministration of cholestyramine resin and the bacterial suspension on the induction of fluid accumulation (Fig. 5C). The preventive effect of cholestyramine resin on fluid accumulation was stronger when added together with the bacterial suspension than when the loops were pretreated with cholestyramine and then inoculated (cf. Fig. 5C with Fig. 5A) and a significant decrease in fluid accumulation was observed even with the highest dose of *V. parahaemolyticus* (Fig. 5C). In contrast, coadministration of Dowex800 400 mesh resin had no effect on fluid accumulation. As a T3SS gene cluster similar to the T3SS2 gene cluster of KP-positive *V. parahaemolyticus* is present in *trh*-positive (KP-negative) *V. parahaemolyticus* and non-O1/non-O139 *V. cholerae* [21], [22], we next examined whether cholestyramine would also prevent the induction of fluid accumulation by these species. For the non-O1/non-O139 *V. cholerae*, we first performed PCR-based genotyping to confirm the presence of T3SS2-related genes (Fig. S5). Fluid accumulation induced by both *trh*-positive *V. parahaemolyticus* and the *V. cholerae* strain was significantly decreased when the bacteria were injected simultaneously with cholestyramine (Fig. 5D). These results indicate that endogenous bile acids in the intestinal tract are at least one of a host-derived inducer for induction of fluid accumulation by T3SS2-positive pathogens and sequestration of bile acids by cholestyramine resin may be useful for preventing diarrhea caused by these bacteria.

## Discussion

T3SS2-related genes encoded in the Vp-PAI region are considered to be involved in the pathogenicity of *V. parahaemolyticus* to humans [18], [20]. We previously showed that two positive regulators, VtrA and VtrB, are essential for expression of these genes and that *vtrA*- and *vtrB*-null mutants lack the capacity to induce fluid accumulation *in vivo* [23]. However, nothing has been known about environmental factors that affect expression of Vp-PAI genes or the host-derived factors that trigger the production of these virulence genes. In this study, we demonstrated that several bile acids in crude bile strongly elevate transcription of Vp-PAI-encoded genes under intestinal osmotic conditions. The Vp-PAI gene-inducing activity of crude bile and bile acids was saturated at concentrations of 0.04% and 100 µM, respectively. Although the bile acid concentration in the intestine varies, it usually ranges from 0.2% to 4% [28]. Therefore, the concentrations of crude bile and bile acids used in this study are representative of concentrations *in vivo*.

The presence of bile in the lumen of the human intestine is necessary for the digestive process [28]. Bile acids, a major component of crude bile, affect the production of the virulence factors of several enteric pathogens [28], [30]. For instance, bile has been shown to reduce the invasion of *Salmonella typhimurium* into eukaryotic cells through transcriptional repression of T3SS genes located in the *Salmonella* pathogenicity island (SPI-1) [31]. In contrast to *Salmonella*, bile induces *Shigella* spp. invasiveness in that bile salts DC and CDC induce secretion of the T3SS protein, Ipa, which promotes the invasion of epithelial cells [32]. In *V. cholerae*, production of the major virulence factors, CT and TCP, was substantially reduced by bile in a ToxT-dependent manner (ToxT is a transcriptional activator of these genes) [33], [34]. Osawa et al. demonstrated that production of TDH by *V. parahaemolyticus* was enhanced by several bile acids [35], [36]. It was also reported that bile and DC increased adherence of both KP-positive and KP-negative *V. parahaemolyticus* to Int-407 cells *in vitro* [37]. Recently, it was reported that bile modulated T3SS genes expression of non-O1/non-O139 *V. cholerae* [38]. As described above, bile is considered closely associated with the production of virulence factors of enteric pathogens. However, the importance and role of bile during the *in vivo* infection process has remained largely unknown. Here, we demonstrated that bile acids are important not only for the production of T3SS2-related proteins and TDH *in vitro* but also for induction of fluid accumulation *in vivo*. To our knowledge, this is the first report showing that bile acid stimulation is actually responsible for the virulence of enteric pathogens *in vivo.* It has been reported that bile acids induce CT expression in *V. cholerae* strain O395 in a ToxRS-dependent and ToxT-independent manner [27]. Vp-ToxR of *V. parahaemolyticus*, a homolog of the *V. cholerae* ToxR, has been reported to be essential for TDH production and for the enterotoxic activities of the AQ3815 strain [39]. In our investigation, bile-induced production of TDH and T3SS2-related proteins and *V. parahaemolyticus*-induced fluid accumulation were dependent on VtrA and VtrB and no participation of Vp-ToxR in these processes was observed (Fig. S2A and B). Therefore, we conclude that Vp-ToxR is not responsible for bile-mediated expression of these virulence factors or for *V. parahaemolyticus*-induced fluid accumulation. However, this conclusion should be validated by studies on other strains of *V. parahaemolyticus*.

It has been demonstrated that host adrenergic agonists such as norepinephrine activate transcription not only of genes encoded by the T3SS gene cluster, but also of flagellar- and shiga-toxin-producing genes [40]. There is an intriguing report that shows that the pathogenicity of *V. parahaemolyticus* is also augmented by norepinephrine [41]. Therefore, we considered the possibility that the suppressive effect of the cholestyramine-treated ileal loop on fluid accumulation was due to absorptive removal of norepinephrine. However, as shown in Fig. S4, exogenous norepinephrine administration did not result in recovery of fluid accumulation in the cholestyramine-treated ileal loop. Norepinephrine does not appear to elevate transcription of T3SS2 genes [41]. Thus, we conclude that norepinephrine removal does not contribute to the attenuating effect of cholestyramine treatment on fluid accumulation and that the effect of norepinephrine on *V. parahaemolyticus*-induced fluid accumulation reported by Nakano et al. occurs via a pathway other than that through which the effects of bile acids are exerted.

In contrast to the effect of crude bile on the production of T3SS2-related proteins and TDH, expression of T3SS1-related proteins was suppressed by bile (Fig. S1A), which was also illustrated by decreased cytotoxic activity against Caco-2 cells (Fig. S1B). However, bile did not significantly affect the level of transcription of these genes according to microarray analysis (Fig. 3A and Table S2), suggesting that a compound (or compounds) in crude bile is involved in posttranscriptional regulation of T3SS1-related protein expression.

This study revealed that 1) *V. parahaemolyticus* recognizes its arrival in the intestine by sensing bile acids, 2) bile acids induce the transcription of Vp-PAI genes via VtrA and VtrB and 3) transduction of this signal facilitates fluid accumulation in the host. This mechanism of bile acid-induced expression of virulence genes in the human intestinal tract appears to be common to T3SS2-positive pathogens because fluid accumulation induced by *trh*-positive *V. parahaemolyticus* and non-O1/non-O139 *V. cholerae* was also inhibited by cholestyramine (Fig. 5). These prevention effects of cholestyramine treatment on fluid accumulation *in vivo* were less effective compared with that of in vitro assays. We predict two possible causes. One possibility is that cholestyramine treatment could not remove endogenous bile salts from the intestinal tract completely. As shown in Fig. 1 and Fig. 4, bile and bile acids can induce expression of Vp-PAI genes at low concentration. Therefore, residual bile acids in intestine might remain to stimulate Vp-PAI genes expression. Another possibility is that a host-derived inducer(s) other than bile acids exists in the intestinal tract. We will plan to explore this issue in our future research. Despite of this, it is amazing that these pathogens, which occur naturally in marine, estuarine or aquatic environments, are equipped with an extremely specialized sensing system for adapting to the human body and causing disease.

Cholestyramine is a bile acid sequestrant that binds bile acids in the gastrointestinal tract, forming an insoluble complex [42], [43]. Such sequestration stimulates the conversion of plasma cholesterol into bile acids to normalize intestinal bile acid levels. As it has very few side effects, cholestyramine is frequently used to treat hypercholesterolemia [43]. Given the increase in numbers of antibiotic-resistant pathogenic bacteria and the emergence of multidrug-resistant strains of vibrios from patients and environmental sources worldwide [44]–[48], drugs targeted at suppressing bacterial virulence mechanisms instead of killing bacteria or inhibiting their growth (the aims of most conventional antibiotics) constitute an alternative approach to treating infections [49]–[51]. Inactivation of bile acids using sequestrants such as cholestyramine exploits a weakness of T3SS2-positive pathogens and represents a potential novel antivirulence therapy that may attenuate the development of drug-resistant bacteria. This new approach warrants further validation.

## Materials and Methods

### Bacterial strains and plasmids

*V. parahaemolyticus* strain RIMD2210633 (KP positive, serotype O3:K6) [17] was used for constructing deletion mutants and for functional analysis. *E. coli* DH5α and SM10λ*pir* were used for general manipulation of plasmids and mobilization of plasmids into *V. parahaemolyticus*. The strains and plasmids used in this study are listed in Table S2.

### Immunoblot analysis

*V. parahaemolyticus* was grown overnight in LB broth containing 3% NaCl at 37°C. For measurement of protein production under various temperature conditions, an overnight culture was diluted 1∶100 into LB with 0.5% NaCl and grown to an OD_600_ of 1.0. For measurement of protein production under various osmotic conditions, an overnight culture was diluted 1∶100 into LB medium containing various concentrations of NaCl and grown to an OD_600_ of 1.0. For crude bile and bile salt induction experiments, cells were grown for 3 h in LB medium containing 0.3 M NaCl with or without crude bile (OX Gall powder, Sigma) or bile salts (Sigma). After incubation, bacterial cultures were centrifuged and bacterial pellets were solubilized using Laemmli buffer. Secreted proteins were harvested by precipitation with trichloroacetic acid (10% v/v) on ice for 60 min, followed by centrifugation at 48,000 *g* for 60 min. The pellets were rinsed in cold acetone and solubilized in Laemmli buffer.

Samples for western blot analysis were separated using SDS PAGE (12.5% polyacrylamide; COSMO BIO). The transferred membranes were probed with anti-VscC1, anti-VopD1, anti-VepA, anti-VscC2, anti-VopD2, anti-VopC, anti-TDH, anti-VtrA or anti-VtrB polyclonal antibodies or with anti-RNA polymerase (RNAP) β-subunit monoclonal antibody (SANTA CRUZ BIOTECHNOLOGY) and then probed with horseradish peroxidase-conjugated goat anti-rabbit antibody (ZYMED). The blots were developed using enhanced chemiluminescence western blotting kits (GE HEALTHCARE).

### Cytotoxicity assay

Cytotoxicity assays were performed as previously described [23]. Briefly, *V. parahaemolyticus* strains were grown in LB medium containing 0.3 M NaCl with or without 0.04% crude bile for 3 h and were washed with PBS. Caco-2 cells were cocultured for 4.5 h with PBS-washed bacteria at a multiplicity of infection of 10. The release of lactate dehydrogenase (LDH) into the medium was quantified using CytoTox96 (Promega). The LDH release (percentage cytotoxicity) was calculated using the following equation: ((OD at 490 nm [OD_490_] of experimental release – OD_490_ of spontaneous release)/(OD_490_ of maximum release – OD_490_ of spontaneous release)) × 100. Spontaneous release was defined as the amount of LDH released from the cytoplasm of uninfected cells, whereas maximum release was defined as the amount of LDH released after total lysis of uninfected cells.

### Microarray experiments

Microarray analyses were performed as previously described [23]. Briefly, *V. parahaemolyticus* strains were grown at 37°C in LB broth containing 0.3 M NaCl to an OD_600_ of 0.6, and were then incubated with or without 0.04% crude bile for 30 min. Bacteria were harvested by centrifugation and highly purified total RNA was finally isolated using QIAGEN RNeasy mini kits according to the manufacturer's protocol. RNA was transcribed to DNA and labeled with aminoallyl dUTP using reverse transcriptase (Superscript III; Invitrogen) and random hexamers (TAKARA Bio) as primers. The aminoallyl-labeled DNA was labeled with Cy3 or Cy5 dye. Cy3- or Cy5-labeled probe mixtures were applied to microarray slides, which were then incubated for 16 h at 55°C in a MAUI hybridization chamber. After washing, the microarray slides were scanned using a Scan Array Express Lite instrument (Perkin Elmer Life and Analytical Sciences). Each experiment was repeated in triplicate. Microarray data were analyzed using ScanArray Express software (Perkin Elmer Life and Analytical Sciences). Data were filtered for statistical significance (P<0.05) using a MultiExperiment Viewer *t* test (MeV, http://www.tm4.org/mev/). The hierarchical clustering of filtered microarray data was performed using the average-linkage method, the euclidean distance metric and MeV. All of gene expression data were MIAME compliant and were deposited in the NCBI Gene Expression Omnibus database (GEO; http://www.ncbi.nlm.nih.gov/geo/) under accession number GSE21666.

### Reporter gene assays

*V. parahaemolyticus* strains harboring a reporter plasmid were grown for 2 h at 37°C in LB broth containing 0.3 M NaCl with or without 0.04% crude bile or various concentrations of bile salts. The enzymatic activity of cell lysates was measured using Miller's method with *o*-nitrophenyl-β-D-galactopyranoside as substrate [52].

### Rabbit ileal loop test

*V. parahaemolyticus* strains were grown overnight in LB broth containing 0.3 M NaCl. Cultures were then diluted 1∶100 into LB broth with 0.3 M NaCl and grown with shaking for 5.5 h. After incubation, bacteria were harvested by centrifugation and suspended in LB broth containing 0.3 M NaCl. To determine the effect of endogenous bile acid depletion from the rabbit small intestine on fluid accumulation, 5% cholestyramine (Sigma) or Dowex800 400 mesh (Sigma) slurry in PBS was passed through the small intestine. After washing with PBS to remove the remaining resin, the bacterial suspensions (10^5^–10^9^ CFU per loop) were injected into the ileal loops. To determine the effect of coexistence of cholestyramine or Dowex800 400 mesh on fluid accumulation, various concentrations (10^6^–10^9^ CFU/ml) of bacteria were suspended in LB broth containing 1% cholestyramine or Dowex800 400 mesh and then injected into the rabbit ileal loops. The fluid accumulation in each loop was measured 16 h after the challenge. The result was expressed as the amount of accumulated fluid (ml) per cm of ligated rabbit small intestine. All animal experiments were performed according to an experimental protocol approved by the Ethics Review Committee for Animal Experimentation of the Research Institute for Microbial Diseases (Osaka University, Osaka, Japan).

### Statistical analysis

All data are presented as the mean ± SD of three determinations per experimental condition. Statistical significance was determined using one-way ANOVA followed by Dunnett's multiple comparison test. P < 0.05 was considered statistically significant.

## Supporting Information

Figure S1 Crude bile represses production of T3SS1-related proteins. A. Effect of crude bile on the production of T3SS1-related proteins by V. parahaemolyticus. Immunoblot analysis of V. parahaemolyticus cultured in LB broth (0.3 M NaCl) at 37°C in the presence of various concentrations of crude bile (0-0.04%). Blots were probed with anti-VopD1 (T3SS1 translocon protein), anti-VepA (T3SS1 effector protein), anti-VscC1 (T3SS1 apparatus protein) and anti-RNAP antibodies. B. Crude bile represses T3SS1-dependent cytotoxicity of V. parahaemolyticus. V. parahaemolyticus strains (ΔvcrD1ΔvcrD2, gray bar; POR-3, black bar) were cultured in LB medium containing 0.3 M NaCl at 37°C in the presence of various concentrations of crude bile (0-0.04%) for 3 h. After incubation, the strains were exposed to Caco-2 cells for 4.5 h. Cytotoxicity was evaluated according to the amount of LDH released. Error bars represent SDs for triplicate independent experiments.
(8.73 MB TIF)

Figure S2 ToxR is not necessary for crude bile-induced TDH and T3SS2-related protein production or for V. parahaemolyticus-induced fluid accumulation. A. Production of TDH and T3SS2-related proteins by the toxR mutant strain in the presence of crude bile. Immunoblot analysis of V. parahaemolyticus strains cultured in LB broth containing 0.3 M NaCl at 37°C with (+) or without (-) 0.04% crude bile. Blots were probed with anti-TDH, anti-VopD2, anti-VopC, anti-VscC2, anti-VtrB, anti-VtrA, and anti-RNAP (RNA polymerase) antibodies. B. Fluid accumulation induced by the toxR mutant strain. Fluid accumulation induced by various doses (106-109 CFU per loop) of the toxR mutant (gray bars) and a high dose (109 CFU per loop) of WTΔvtrA (light blue bar) or WTΔvtrB (orange bar) were compared with that of fluid accumulation in the presence of the WT (black bars). Data are expressed as the amount of accumulated fluid (ml) per cm of ligated rabbit small intestine. Error bars represent SDs for experiments conducted in sextuplicate.
(9.39 MB TIF)

Figure S3 Production of TDH and T3SS2-related proteins in various concentrations of bile salts. Immunoblot analysis of bacterial whole cell pellets from V. parahaemolyticus cultured in LB medium containing 0.3 M NaCl at 37°C in the presence of various concentrations (0.1-1000 μM) of bile salts (taurodeoxycholate, TDC; glycodeoxycholate, GDC; deoxycholate, DC; taurochenodeoxycholate, TCDC; glycochenodeoxycholate, GCDC; taurocholate, TCA; glycocholate, GCA). Blots were probed with anti-TDH (A), anti-VopD2 (B), anti-VopC (C), anti-VscC2 (D) or anti-RNAP (E) antibodies. F: Immunoblot analysis of bacterial whole cell pellets from V. parahaemolyticus cultured with or without 1000 μM CA, CDC or 0.04% crude bile. Blots were probed with anti-TDH, anti-VopD2, anti-VopC, anti-VscC2 or anti-RNAP antibodies.
(10.15 MB TIF)

Figure S4 The attenuating effect of cholestyramine resin treatment on fluid accumulation is not due to absorptive removal of norepinephrine. V. parahaemolyticus (107 CFU) were suspended in LB medium with or without 100 μM of norepinephrine (NE) and injected into nontreated or cholestyramine-treated ileal loops. Fluid accumulation in each loop was measured 16 h after infection. Error bars represent SDs for experiments conducted in sextuplicate (n.s., not significant).
(6.52 MB TIF)

Figure S5 PCR-based genotyping of non-O1/non-O139 V. cholerae. PCR assays of O1 V. cholerae RIMD2203294 (lane1) and non-O1/non-O139 V. cholerae RIMD2214243 (lane 2) strains were performed to test for the presence of several known virulence genes (vtrA, vtrB, vscN2, vscV2, ctxA, tcpA, hlyA and rtxA). The PCR products were electrophoresed in a 2% agarose gel and were visualized by staining with ethidium bromide. The presence of each gene was also confirmed by direct DNA sequencing of each amplified DNA fragment.
(6.70 MB TIF)

Table S1 Microarray analysis of crude bile responsible genes in V. parahaemolyticus.
(0.12 MB DOC)

Table S2 Strains and plasmids used in this study.
(0.07 MB DOC)
