# Supplementary figures and images for: Bile Acid-Induced Virulence Gene Expression of Vibrio parahaemolyticus Reveals a Novel Therapeutic Potential for Bile Acid Sequestrants
Source: PLoS One. 2010 Oct 13;5(10):e13365. doi: 10.1371/journal.pone.0013365 (PMC2954181; doi:10.1371/journal.pone.0013365)

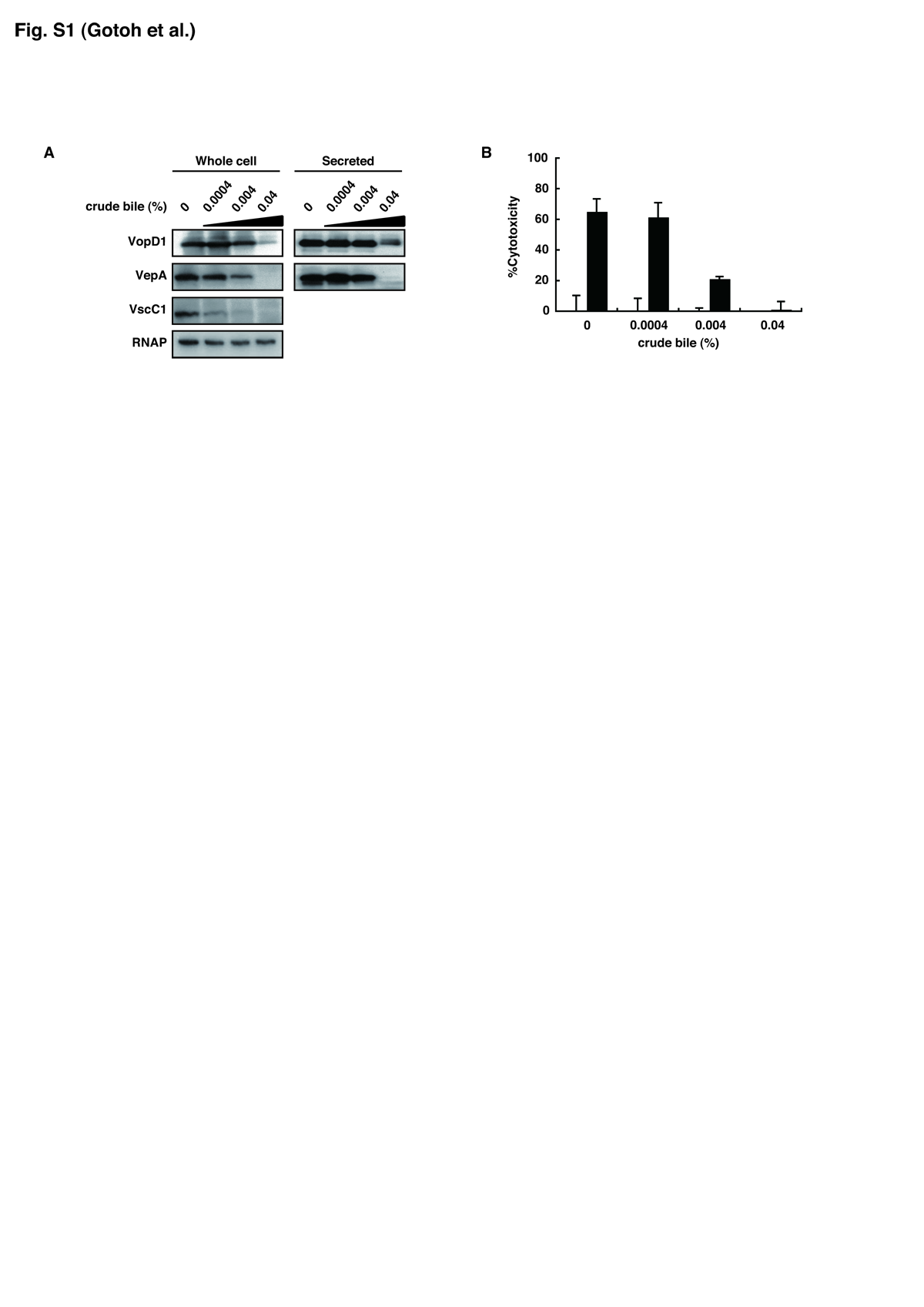

Supplement: Figure S1 — Crude bile represses production of T3SS1-related proteins. A. Effect of crude bile on the production of T3SS1-related proteins by V. parahaemolyticus. Immunoblot analysis of V. parahaemolyticus cultured in LB broth (0.3 M NaCl) at 37°C in the presence of various concentrations of crude bile (0-0.04%). Blots were probed with anti-VopD1 (T3SS1 translocon protein), anti-VepA (T3SS1 effector protein), anti-VscC1 (T3SS1 apparatus protein) and anti-RNAP antibodies. B. Crude bile represses T3SS1-dependent cytotoxicity of V. parahaemolyticus. V. parahaemolyticus strains (ΔvcrD1ΔvcrD2, gray bar; POR-3, black bar) were cultured in LB medium containing 0.3 M NaCl at 37°C in the presence of various concentrations of crude bile (0-0.04%) for 3 h. After incubation, the strains were exposed to Caco-2 cells for 4.5 h. Cytotoxicity was evaluated according to the amount of LDH released. Error bars represent SDs for triplicate independent experiments. (8.73 MB TIF) [file pone.0013365.s001.tif]

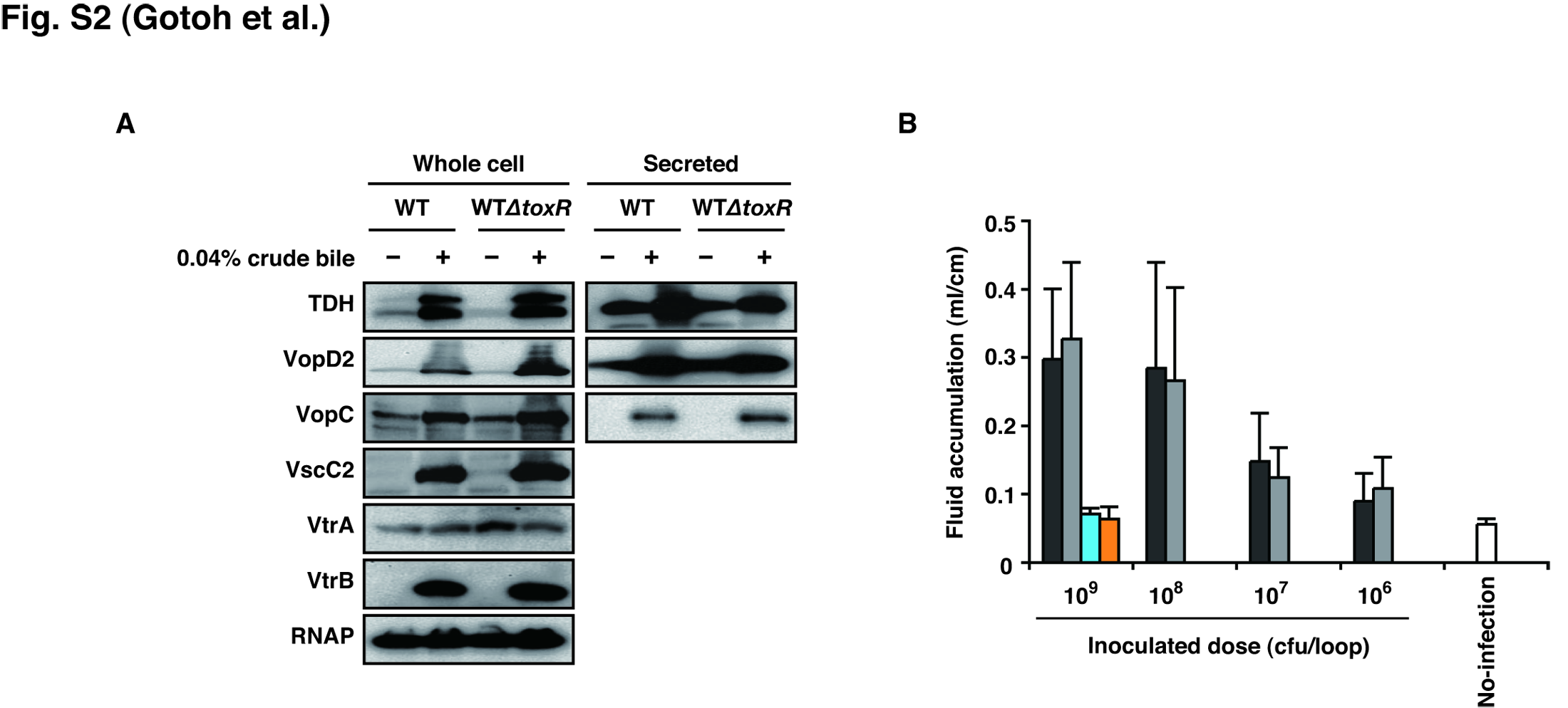

Supplement: Figure S2 — ToxR is not necessary for crude bile-induced TDH and T3SS2-related protein production or for V. parahaemolyticus-induced fluid accumulation. A. Production of TDH and T3SS2-related proteins by the toxR mutant strain in the presence of crude bile. Immunoblot analysis of V. parahaemolyticus strains cultured in LB broth containing 0.3 M NaCl at 37°C with (+) or without (-) 0.04% crude bile. Blots were probed with anti-TDH, anti-VopD2, anti-VopC, anti-VscC2, anti-VtrB, anti-VtrA, and anti-RNAP (RNA polymerase) antibodies. B. Fluid accumulation induced by the toxR mutant strain. Fluid accumulation induced by various doses (106-109 CFU per loop) of the toxR mutant (gray bars) and a high dose (109 CFU per loop) of WTΔvtrA (light blue bar) or WTΔvtrB (orange bar) were compared with that of fluid accumulation in the presence of the WT (black bars). Data are expressed as the amount of accumulated fluid (ml) per cm of ligated rabbit small intestine. Error bars represent SDs for experiments conducted in sextuplicate. (9.39 MB TIF) [file pone.0013365.s002.tif]

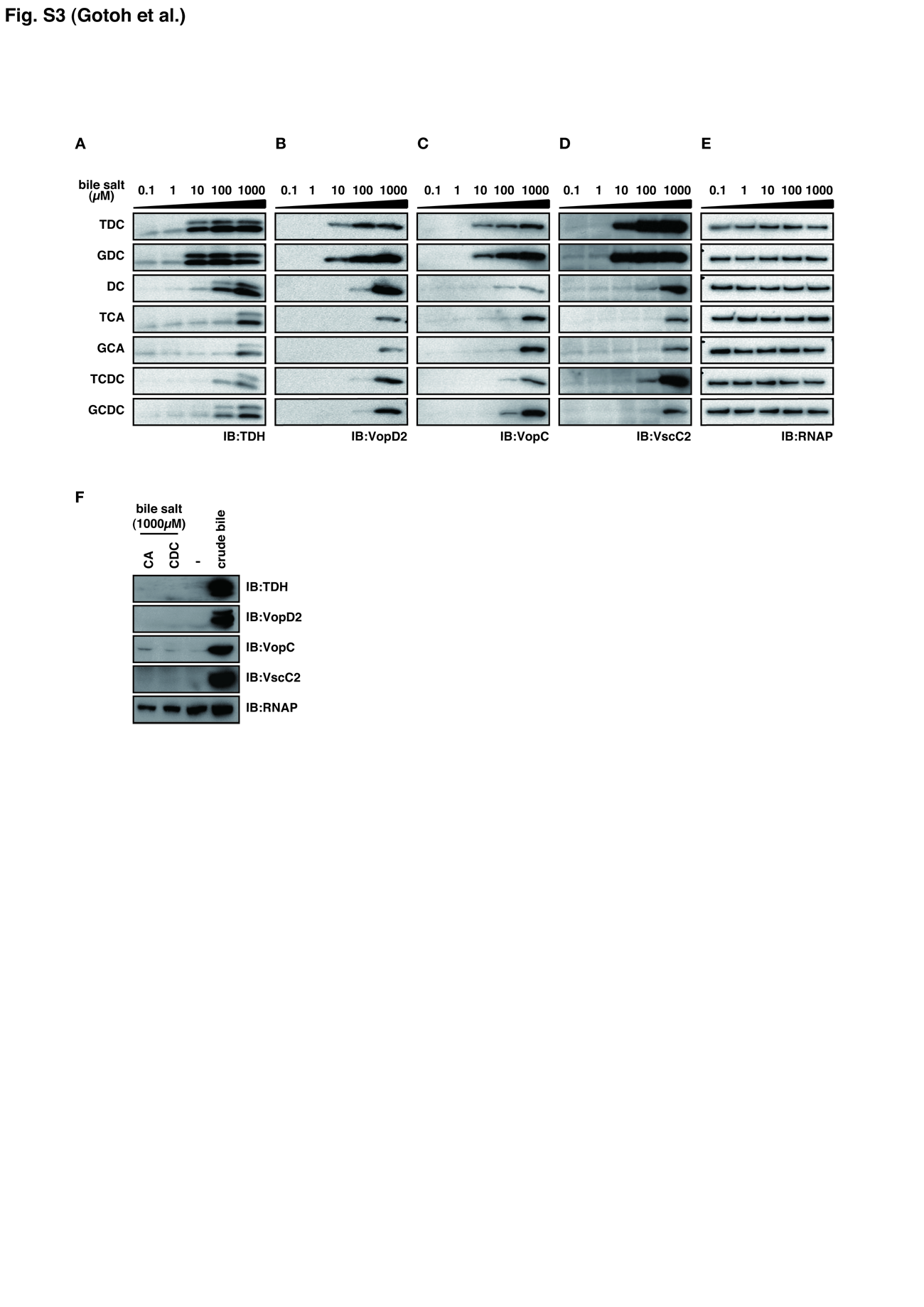

Supplement: Figure S3 — Production of TDH and T3SS2-related proteins in various concentrations of bile salts. Immunoblot analysis of bacterial whole cell pellets from V. parahaemolyticus cultured in LB medium containing 0.3 M NaCl at 37°C in the presence of various concentrations (0.1-1000 μM) of bile salts (taurodeoxycholate, TDC; glycodeoxycholate, GDC; deoxycholate, DC; taurochenodeoxycholate, TCDC; glycochenodeoxycholate, GCDC; taurocholate, TCA; glycocholate, GCA). Blots were probed with anti-TDH (A), anti-VopD2 (B), anti-VopC (C), anti-VscC2 (D) or anti-RNAP (E) antibodies. F: Immunoblot analysis of bacterial whole cell pellets from V. parahaemolyticus cultured with or without 1000 μM CA, CDC or 0.04% crude bile. Blots were probed with anti-TDH, anti-VopD2, anti-VopC, anti-VscC2 or anti-RNAP antibodies. (10.15 MB TIF) [file pone.0013365.s003.tif]

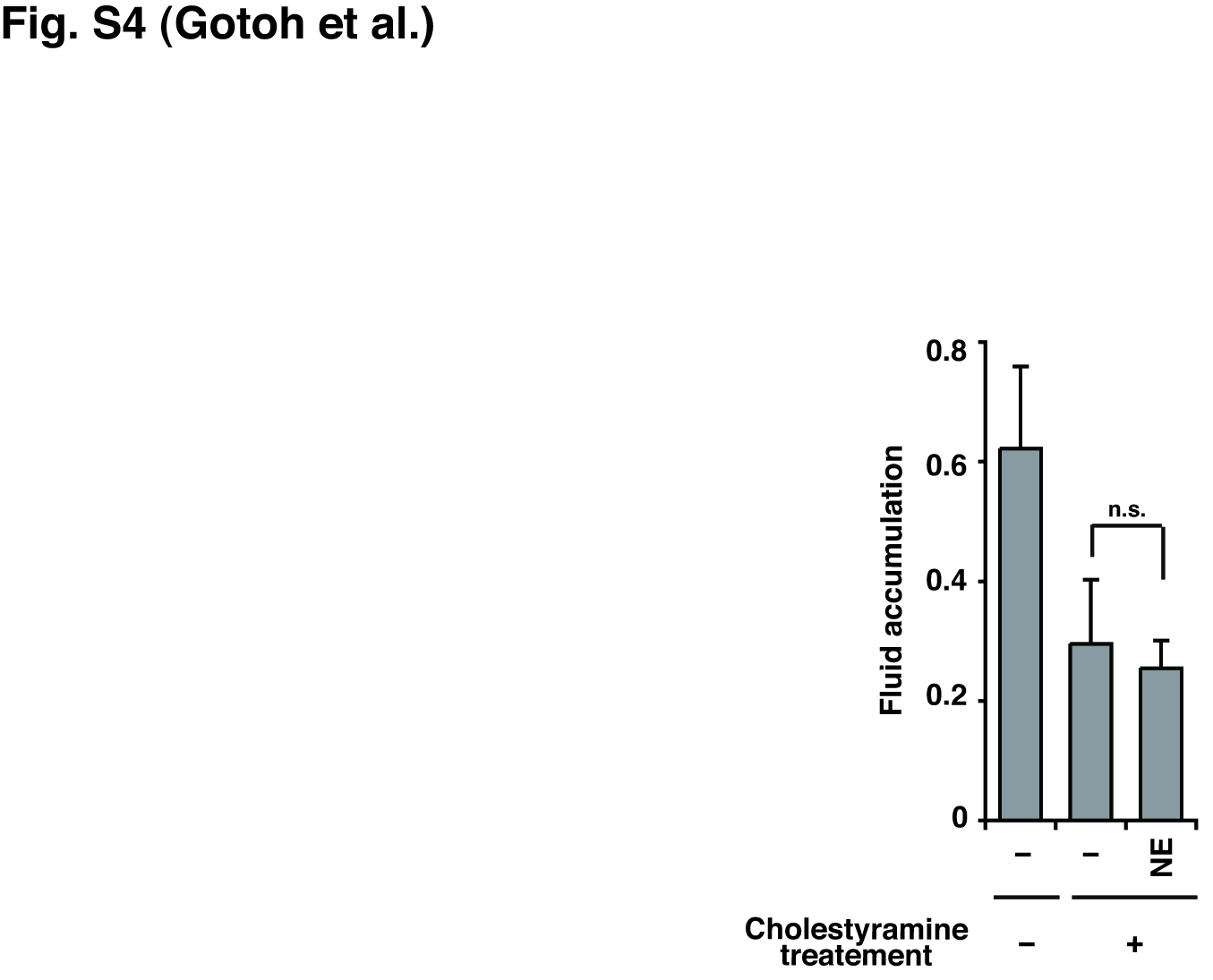

Supplement: Figure S4 — The attenuating effect of cholestyramine resin treatment on fluid accumulation is not due to absorptive removal of norepinephrine. V. parahaemolyticus (107 CFU) were suspended in LB medium with or without 100 μM of norepinephrine (NE) and injected into nontreated or cholestyramine-treated ileal loops. Fluid accumulation in each loop was measured 16 h after infection. Error bars represent SDs for experiments conducted in sextuplicate (n.s., not significant). (6.52 MB TIF) [file pone.0013365.s004.tif]

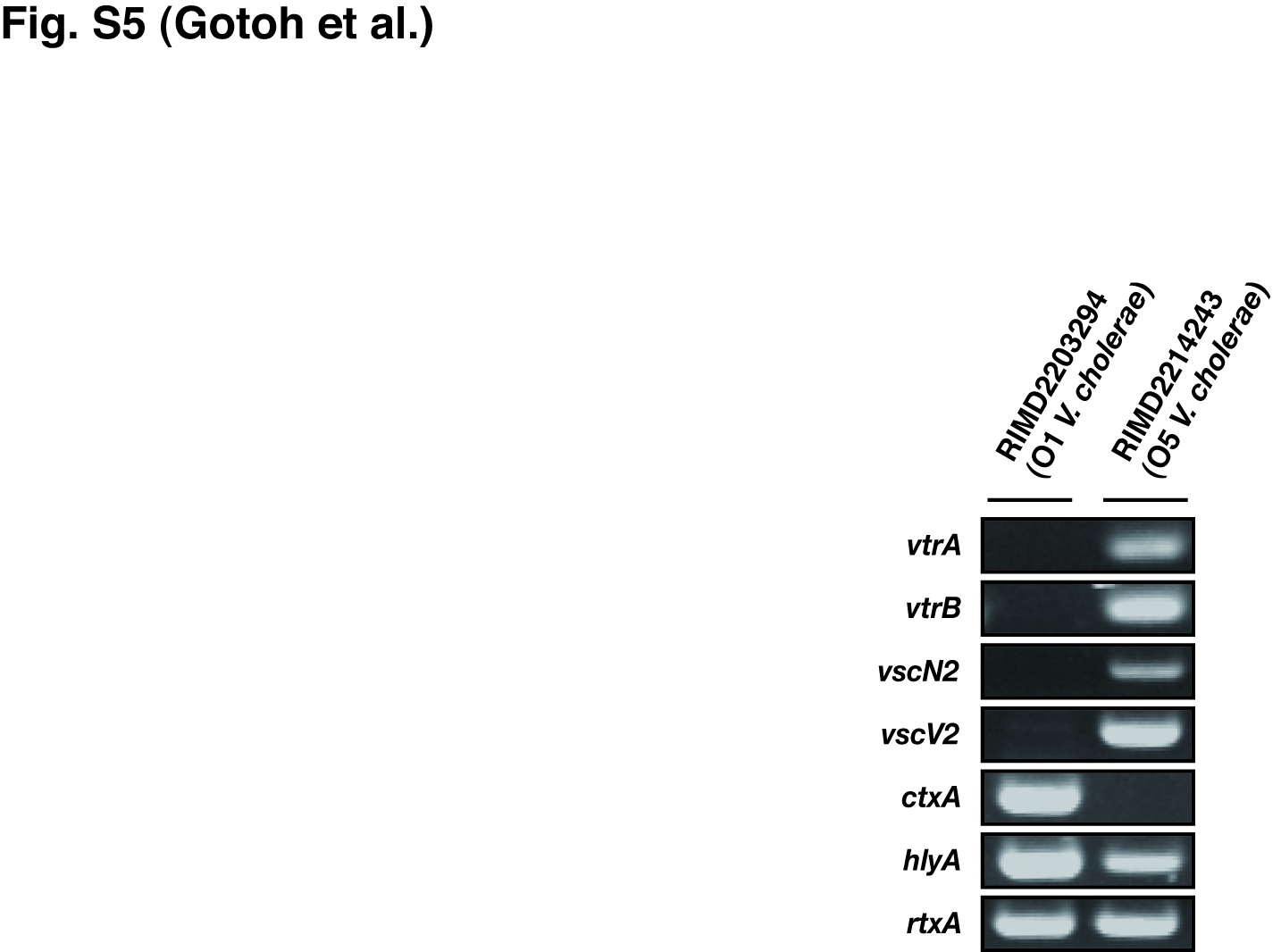

Supplement: Figure S5 — PCR-based genotyping of non-O1/non-O139 V. cholerae. PCR assays of O1 V. cholerae RIMD2203294 (lane1) and non-O1/non-O139 V. cholerae RIMD2214243 (lane 2) strains were performed to test for the presence of several known virulence genes (vtrA, vtrB, vscN2, vscV2, ctxA, tcpA, hlyA and rtxA). The PCR products were electrophoresed in a 2% agarose gel and were visualized by staining with ethidium bromide. The presence of each gene was also confirmed by direct DNA sequencing of each amplified DNA fragment. (6.70 MB TIF) [file pone.0013365.s005.tif]
