# Supplementary material for: Bile Acid-Induced Virulence Gene Expression of Vibrio parahaemolyticus Reveals a Novel Therapeutic Potential for Bile Acid Sequestrants
Source: PLoS One. 2010 Oct 13;5(10):e13365. doi: 10.1371/journal.pone.0013365 (PMC2954181; doi:10.1371/journal.pone.0013365)
Supplement: Table S1 — Microarray analysis of crude bile responsible genes in V. parahaemolyticus. (0.12 MB DOC) [file pone.0013365.s006.doc]

**Supplemental Tables**

**Table S1. Microarray analysis of crude bile responsible genes in *V. parahaemolyticus*.**

| Identification | ORF Description | Fold change^a^ | |
| --- | --- | --- | --- |
|  |  | WT | *∆vtrA* |
| VP0029 | fatty oxidation complex, beta subunit | 4.9 | 5.3 |
| VP0030 | fatty oxidation complex, alpha subunit | 6.2 | 5.3 |
| VP1272 | hypothetical protein | -5.5 | -6.3 |
| VP1713 | conserved hypothetical protein | 10.9 | 8.8 |
| VP1714 | conserved hypothetical protein | 16.1 | 6.3 |
| VP1715 | conserved hypothetical protein | 12.6 | 6.9 |
| VP1717 | conserved hypothetical protein | 10.6 | 10.5 |
| VP1736 | conserved hypothetical protein | -4.1 | -3.7 |
| VP1994 | putative isochorismatase-related protein | -4.1 | -8.4 |
| VP1995 | ABC transporter, ATP-binding protein | -4.7 | -4.6 |
| VP1996 | conserved hypothetical protein | -4.2 | -7.2 |
| VP1997 | conserved hypothetical protein | -4.9 | -5.2 |
| VP1998 | putative outer membrane protein TolC | -4.6 | -8.3 |
| VP1999 | conserved hypothetical protein | -5.5 | -6.5 |
| VP2194 | 3-oxoacyl-[acyl-carrier-protein] synthase I | -10.7 | -14.4 |
| VP2209 | fatty oxidation complex, beta subunit | 4.0 | 5.1 |
| VP2509 | lipid A biosynthesis lauroyl acyltransferase | -5.0 | -5.5 |
| VPA0226 | lecithin-dependent hemolysin (LDH) | 5.8 | 7.1 |
| VPA0318 | putative outer membrane protein OmpV | -4.3 | -4.2 |
| VPA0319 | hypothetical protein | -4.0 | -3.3 |
| VPA0375 | putative adenylosuccinate synthase | 4.3 | 3.3 |
| VPA0603 | NodN-related protein | 5.2 | 4.5 |
| VPA1279 | diacylglycerol kinase | -3.8 | -4.0 |
| **VPA1311** | hypothetical protein | 4.6 | nc |
| **VPA1313** | hypothetical protein | 5.1 | nc |
| **VPA1314** | thermostable direct hemolysin A | 11.8 | nc |
| **VPA1315** | hypothetical protein | 4.1 | nc |
| **VPA1321** | VopC; T3SS2 effector protein | 4.3 | nc |
| **VPA1322** | putative zinc finger protein | 4.3 | nc |
| **VPA1323** | hypothetical protein | 5.8 | nc |
| **VPA1324** | hypothetical protein | 50.4 | nc |
| **VPA1327** | VopT; T3SS2 effector protein | 5.4 | nc |
| **VPA1328** | hypothetical protein | 10.2 | nc |
| **VPA1329** | putative traA protein | 13.4 | nc |
| **VPA1330** | hypothetical protein | 7.5 | nc |
| **VPA1331** | putative OspC2 | 9.3 | nc |
| **VPA1334** | hypothetical protein | 9.9 | nc |
| **VPA1335** | putative type III secretion apparatus protein | 5.1 | nc |
| **VPA1336** | hypothetical protein | 9.5 | nc |
| **VPA1337** | hypothetical protein | 9.1 | nc |
| **VPA1338** | VdcN2 | 12.5 | nc |
| **VPA1339** | VscC2 | 38.9 | nc |
| **VPA1340** | hypothetical protein | 37.9 | nc |
| **VPA1341** | putative Spa29, component of the Mxi-Spa secretion machinery | 32.2 | nc |
| **VPA1342** | putative Type III secretion protein Spa24 | 45.1 | nc |
| **VPA1343** | hypothetical protein | 58.4 | nc |
| **VPA1344** | hypothetical protein | 10.2 | nc |
| **VPA1345** | hypothetical protein | 23.5 | nc |
| **VPA1346** | VopP; T3SS2 effector protein | 35.5 | nc |
| **VPA1347** | hypothetical protein | 10.6 | nc |
| **VPA1348** | VtrB | 12.8 | nc |
| **VPA1349** | putative Type III secretion protein Spa33 | 14.1 | nc |
| **VPA1350** | hypothetical protein | 19.9 | nc |
| **VPA1351** | hypothetical protein | 22.2 | nc |
| **VPA1352** | hypothetical protein | 26.1 | nc |
| **VPA1353** | putative outer membrane protein | 28.1 | nc |
| **VPA1354** | putative type III secretion system EscU protein | 43.7 | nc |
| **VPA1355** | putative type III secretion system EscV protein | 27.7 | nc |
| **VPA1357** | hypothetical protein | 6.8 | nc |
| **VPA1358** | putative dimethyladenosine transferase | 17.5 | nc |
| **VPA1359** | hypothetical protein | 19.6 | nc |
| **VPA1360** | hypothetical protein | 19.3 | nc |
| **VPA1361** | VopD2; T3SS2 translocon protein | 27.2 | nc |
| **VPA1362** | VopB2; T3SS2 translocon protein | 21.6 | nc |
| **VPA1363** | putative chaperone | 47.9 | nc |
| **VPA1364** | hypothetical protein | 34.4 | nc |
| **VPA1365** | putative two-component response regulator | 25.6 | nc |
| **VPA1366** | hypothetical protein | 38.5 | nc |
| **VPA1367** | putative type III secretion system lipoprotein precursor EprK | 34.2 | nc |
| **VPA1368** | hypothetical protein | 54.2 | nc |
| **VPA1370** | VopL; T3SS2 effector protein | 37.3 | nc |
| **VPA1371** | hypothetical protein | 7.0 | nc |
| **VPA1372** | hypothetical protein | 11.1 | nc |
| **VPA1373** | hypothetical protein | 23.2 | nc |
| **VPA1376** | conserved hypothetical protein | 26.8 | nc |
| **VPA1378** | thermostable direct hemolysin S | 9.8 | nc |
| **VPA1380** | putative OspB protein | 12.7 | nc |

^a^ Fold change (≥ 4-fold difference with P < 0.05) in gene transcripts between the wild-type in the absence of crude bile and the wild-type or *∆vtrA* in the presence of crude bile as determined microarray analysis. Nc indicates not change. Vp-PAI encoded genes are highlighted in bold.
