# Supplementary material for: Bile Acid-Induced Virulence Gene Expression of Vibrio parahaemolyticus Reveals a Novel Therapeutic Potential for Bile Acid Sequestrants
Source: PLoS One. 2010 Oct 13;5(10):e13365. doi: 10.1371/journal.pone.0013365 (PMC2954181; doi:10.1371/journal.pone.0013365)
Supplement: Table S2 — Strains and plasmids used in this study. (0.07 MB DOC) [file pone.0013365.s007.doc]

**Table S2**. Strains and plasmids used in this study

| Strains and plasmid | Description | Source or reference |
| --- | --- | --- |
| ***Vibrio parahaemolyticus*** |  |  |
| WT | RIMD2210633 (KP positive, serotype O3:K6) | [17] |
| POR-1 | *∆tdhAS* derivative of WT | [53] |
| POR-2 | POR-1 knockout of *vcrD1* (*vp1696*) gene | [53] |
| POR-3 | POR-1 knockout of *vcrD2* (*vpa1355*) gene | [53] |
| *∆vcrD1∆vcrD2* | POR-1 knockout of *vcrD1* and *vcrD2* gene | [26] |
| WT*∆vtrA* | KXV237 knockout of *vtrA* (*vp1332*) gene | [24] |
| WT*∆vtrB* | KXV237 knockout of *vtrB* (*vp1348*) gene | [24] |
| WT*∆vtrA ∆vtrB* | KXV237 knockout of *vtrA* and *vtrB* gene | [24] |
| WT*∆toxR* | KXV237 knockout of *toxR* (*vp0820*) gene | This study |
| TH3996 | Clinical isolate, *trh*^+^ *ure*^+^ *tdh*^-^ | [54] |
|  |  |  |
| ***Vibrio cholerae*** |  |  |
| RIMD2203294 | Clinical isolate, serotype O1 ogawa, *ctx*^+^ | This study |
| RIMD2214243 | Clinical isolate, serotype O5, *ctx*^-^ | This study |
|  |  |  |
| ***Escherichia coli*** |  |  |
| DH5α | F^-^ F80∆*lac*ZM15 *∆* (*lacZYA argF*)*U169* *deoP* *recA1* *endA1* *hsdR17* (r_K_^-^ m_K_^-^) | Laboratory collection |
| SM10l*pir* | *thi thr leu tonA* *lacY* *supE* *recA*::RP4-2-Tc::Mu l*pir* R6K |  |
|  |  |  |
|  |  |  |
| **Plasmid** |  |  |
| pHRP309 | *lacZ* transcriptional fusion vector, Gm^r^ | [55] |
| p309-Pro-*vtrA* | Derivative of pHRP309, containing *vtrA* promoter | [24] |
| p309-Pro-*vtrB* | Derivative of pHRP309, containing *vtrB* promoter | [24] |
| pYAK1 | R6K-*ori* suicide vector containing *sacB* gene | [56] |
| pYAK1-*∆toxR* | Derivative of suicide vector pYAK1 for generating the *toxR* deletion mutants | This study |
